# Synergistic effects of cardiovascular health and social isolation on adverse pregnancy outcomes

**DOI:** 10.1038/s41598-025-03652-x

**Published:** 2025-05-29

**Authors:** Hisashi Ohseto, Mami Ishikuro, Geng Chen, Ippei Takahashi, Genki Shinoda, Aoi Noda, Keiko Murakami, Masatsugu Orui, Noriyuki Iwama, Masahiro Kikuya, Hirohito Metoki, Atsushi Hozawa, Taku Obara, Shinichi Kuriyama

**Affiliations:** 1 Graduate School of Medicine, Tohoku University, Sendai, Miyagi Japan; 2 Tohoku Medical Megabank Organization, Tohoku University, 2-1, Seiryo-machi, Aoba-ku, Sendai, Miyagi 980-8573 Japan; 3 Tohoku University Hospital, Tohoku University, Sendai, Miyagi Japan; 4 Graduate School of Medicine, Teikyo University, Itabashi-ku, Tokyo Japan; 5 Graduate School of Medicine, Tohoku Medical and Pharmaceutical University, Sendai, Miyagi Japan; 6 International Research Institute of Disaster Science, Tohoku University, Sendai, Miyagi Japan

**Keywords:** Cohort study, Health disparities, Life’s Essential 8, Prenatal care, Cardiovascular diseases, Reproductive disorders, Epidemiology, Disease prevention, Public health

## Abstract

Adverse pregnancy outcomes (APOs) affect approximately 20% pregnant women, and their incidence is increasing. The aim of this study was to investigate the effect of cardiovascular health (CVH) during pregnancy on APOs. We analyzed data from 14,930 pregnant women in the Tohoku Medical Megabank Project Birth and Three-Generation Cohort Study. CVH status during pregnancy was assessed using the eight components of Life’s Essential 8. APOs were defined as composite outcomes encompassing preeclampsia, gestational diabetes mellitus, preterm birth, and small for gestational age. The numbers of participants with high, moderate, and low CVH status were 2891 (19.4%), 11,498 (77.0%), and 541 (3.6%), respectively. Poisson regression analyses with robust error variance, which adjusted for maternal age at conception, alcohol consumption, conception via in vitro fertilization, parity, psychological distress, social isolation, and household income, showed a positive association between moderate and low CVH levels and APOs (risk ratio and 95% confidence interval 1.15 [1.03–1.28] and 2.14 [1.78–2.58], respectively). Among pregnant women with low CVH, those who reported social isolation had a higher prevalence of APOs than did those without social isolation (36.4% vs. 27.4%). This difference was attenuated for pregnant women with high CVH status (13.6% vs. 13.1%). In conclusion, CVH status may be useful for assessing the risk of APOs. Socially isolated pregnant women are more vulnerable to the effects of low CVH status.

**Supplementary Information:**

The online version contains supplementary material available at 10.1038/s41598-025-03652-x.

## Introduction

Adverse pregnancy outcomes (APOs), which encompass unfavorable events or complications occurring during pregnancy or delivery, or in the postpartum period, affect approximately 20% pregnant women and are increasing^1,2^. Following delivery, APOs can progress to cardiovascular disease (CVD)^3–5^ and mortality^6^. Placental formation and cardiometabolic factors play crucial roles in APO development^7,8^, and their pathogenic similarity to CVDs has led to the characterization of pregnancy as a “stress test for CVD”^9^. Risk factors for APOs, such as obesity^10^, poor sleep quality^11^, and poor dietary habits^12^ are also recognized as risk factors for CVD^13^. Therefore, it is expected that similar preventive strategies employed for CVD can be applied to APOs.

In 2022, Life’s Essential 8 (LE8) was proposed by the American Heart Association^13^ as an updated framework for assessing cardiovascular health (CVH) to promote disease prevention and health. This concept emphasizes individual health while addressing existing CVD and associated risk factors. LE8 serves as an operational metric for CVH that includes eight components: diet, physical activity (PA), nicotine exposure, sleep health, body mass index (BMI), blood lipids, blood glucose, and blood pressure (BP). Unlike Life’s Simple 7 (LS7), a prior version proposed to assess CVH metrics in 2010^13^, LE8 includes sleep health, which was introduced as a new component in 2022, and all eight CVH components have been rescaled to continuous variables. To date, over 2500 studies have referenced original articles describing LS7^13^, and evidence on LE8 is growing^14,15^. A meta-analysis revealed associations of CVH with CVD incidence, CVD mortality, and all-cause mortality^16^. Furthermore, these associations have been observed in adolescents and young adults^17^. Therefore, we hypothesized that LE8 may be relevant for a wide range of APOs because of shared characteristics between CVD and APOs.

The integration of LE8 into antenatal care could enhance maternal health. Many previous studies solely quantified the impact of a single component of CVH, neglecting the complex interconnections of various components. LE8 provides a comprehensive overview of maternal CVH during pregnancy and is useful for assessment in clinical scenarios where a single factor (e.g., smoking cessation) is related to others (e.g., weight gain or poor dietary habits)^18^. One study^19^ exploring the link between LS7 and APOs demonstrated that improved CVH was associated with better pregnancy outcomes. Notably, this study utilized only five of the seven components, and it remains unclear whether a comprehensive CVH assessment using LE8 is beneficial in antenatal care. In 2024, a study examining the association of LE8 with offspring outcomes was published for the first time^20^. The main findings of that study pertained to neonatal anthropometric measures, demonstrating that better maternal CVH was associated with a lower risk of large for gestational age (LGA) and macrosomia. However, it did not investigate key perinatal complications such as preeclampsia (PE) and gestational diabetes mellitus (GDM). Furthermore, the clinical significance of psychological health and the social determinants involved in effect modification cannot be overlooked. They have a solid clinical basis and underpin all the CVH metrics, interacting bidirectionally^13,21,22^. Previous research has revealed that poor CVH is related to depressive symptoms^23,24^ and social isolation^25^. Another study found an effect modification by CVH on the relationship between socioeconomic status and life expectancy^21^. To date, no study has investigated effect modification by psychological health and social determinants on the relationship between CVH and APOs.

The aim of this study was to investigate the effect of CVH assessed using LE8 on APOs in 14,930 pregnant Japanese women. We also explored the effect modification by psychological distress, social isolation, and income.

## Materials and methods

### Participants

This was a prospective cohort study. Between 2013 and 2017, the Tohoku Medical Megabank Project Birth and Three-Generation (TMM BirThree) Cohort Study^26,27^ recruited 23,406 pregnant women from more than 50 obstetric clinics and hospitals in Miyagi Prefecture, Japan. Of the 23,406 recruited participants, 232 withdrew consent. We then excluded 4,233 participants, including those with multiple births (n = 313), those who delivered before or at 32 weeks of gestation (n = 223), those who had their blood samples drawn after 32 weeks of gestation (n = 3,724), and those with missing data on delivery status (n = 965). In this study, we focused on women who remained pregnant at 32 weeks and had completed blood sampling by that time, to ensure a consistent temporal relationship between CVH assessment and outcomes. The cutoff of 32 weeks of gestation was chosen to capture cardiovascular changes on transition from the second to the third trimester and to prevent pregnancy complications from affecting the CVH status. Additionally, this cutoff value aligned with those in previous studies^19,28^. Participants who were enrolled multiple times in the TMM BirThree Cohort Study were identified, and only their first valid data were used in this study (n = 620). Finally, participants with missing CVH metrics and covariates were excluded (n = 3,391), leaving 14,930 participants for the main analysis. The study flowchart is detailed in Supplementary Fig. 1. Ethical approval was obtained from the Ethics Committee of the Tohoku Medical Megabank Organization (2013-1-103-1), and all participants provided written informed consent for study participation. This study was conducted in accordance with the Declaration of Helsinki.

### CVH definitions

Dietary quality was assessed using the 8-item Japanese Diet Index, which assesses adherence to the Japanese diet and is related to CVD mortality in the Japanese population^29,30^. Food intake was estimated using a food frequency questionnaire^31,32^. PA, nicotine exposure, and sleep health were assessed during pregnancy using self-reported questionnaires. Pre-pregnancy BMI and BP before 20 weeks of gestation were obtained from medical records during antenatal care. Blood lipid and glucose levels before or at 32 weeks of gestation were determined from blood samples collected in the TMM BirThree Cohort Study^26^. Each LE8 component was scored on a scale from 0 (least healthy) to 100 (most healthy), with detailed information provided in Supplementary Table 1. The overall CVH score was the unweighted average of all eight components^13^. Overall and component CVH scores were used to categorize CVH levels as high (80–100), moderate (50–79), and low (0–49), as outlined in the original article^13^.

### Outcomes

APOs were defined as composite outcomes comprising PE, GDM, preterm birth (PTB), and small for gestational age (SGA), based on their pathophysiological relevance to CVH and their clinical impact^5,33^. A diagnosis of PE was established based on the American College of Obstetricians and Gynecologists guidelines^34^ and identified using an algorithm to estimate hypertensive disorders of pregnancy phenotypes from antenatal care records^35,36^. GDM was confirmed through its documentation in medical records at delivery. PTB was defined as birth before 37 weeks of gestation. SGA was defined as birth weight ≤ 10th percentile, according to gestational age-specific birth weight percentiles for the Japanese population. Additional neonatal outcomes, including low birth weight (LBW), LGA, and neonatal intensive care unit (NICU) admission, were identified from medical records. LBW was defined as birth weight < 2500 g, and LGA was defined as birth weight ≥ 90th percentile according to gestational age-specific birth weight percentiles for the Japanese population. NICU admission was confirmed through its documentation in medical records. Other severe conditions, such as eclampsia, were not included as outcomes because of limited data availability, relatively low prevalence, and the fact that they were typically caused by a progression of the more common conditions included as outcomes in this study.

### Covariates

Covariates included maternal age at conception (≥ 35 years old or not), alcohol consumption during pregnancy, conception via in vitro fertilization (IVF), parity (nullipara or not), psychological distress during pregnancy, social isolation during pregnancy, and total annual household income (≤ 4 million yen or not). Age at conception and parity were obtained from medical records during antenatal care, while the remaining variables were self-reported during pregnancy. Psychological distress was evaluated using the Kessler Psychological Distress Scale (K6)^37^, comprising six items rated on a 5-point Likert scale. The total score ranged from 0 to 24, with a score of ≥ 9 indicating psychological distress. Social isolation was evaluated using the Lubben Social Network Scale (LSNS-6)^38^, which comprises six items rated on a 6-point Likert scale. The total score ranges from 0 to 30, with a score of ≤ 11 indicating social isolation. In addition, the LSNS-6 has two subscales: family and friends. Each subscale score ranges from 0 to 15, and a score of ≤ 5 indicates social isolation (Supplementary Table 2)^38^.

### Statistical analysis

Baseline characteristics, CVH scores, and study outcomes were compared according to overall CVH levels and APO status. Continuous variables were analyzed using analysis of variance or Student’s t-test, while categorical variables were analyzed using the chi-square test. Pearson’s correlation coefficients were calculated for all combinations of component CVH scores.

The associations of overall and component CVH levels with study outcomes was explored using Poisson regression with robust error variance to estimate risk ratios (RRs), with high CVH levels used as a reference and adjustment for covariates. Interactions between overall CVH levels and psychological distress, social isolation, and income levels were examined. Given that a potential interaction was observed with social isolation, further subgroup analyses were conducted for the subscales and each item of the LSNS-6 (Supplementary Table 2).

Sensitivity analyses were performed to assess the robustness of the results. First, due to some missing values not satisfying the “missing completely at random” assumption^39^, we conducted association analyses after imputing missing values for CVH metrics and covariates using multiple imputation by chain equations with five iterations^40^ in a population of 18,321 eligible participants. Second, to rule out the potential of reverse causality between CVH and APOs and to account for the clinical significance of very early deliveries, association analyses were conducted solely on participants whose blood samples were collected before or at 20 weeks of gestation (Supplementary Fig. 2). Third, we excluded participants with pre-pregnancy chronic hypertension (CH) (n = 579) or pre-pregnancy diabetes mellitus (DM) (n = 71) from the main analysis. This exclusion aimed to prevent an overestimation of the effect of overall CVH on APOs, given the high risk of PE among patients with pre-pregnancy CH and the consequent diagnosis of GDM for those with pre-pregnancy DM. This approach helps avoid bias in effect estimation and allows for a more homogeneous population, ensuring a clearer interpretation of the association between CVH and APOs.

A threshold P-value of < 0.05 was adopted for detecting statistical significance in the association analyses, while a significance level of *P* < 0.2 was applied for the interaction term. All analyses were conducted using the R software version 4.1.2.

### Paper presentation information

This manuscript has not been published in any journal or presented at any conference in part or in entirety and is not under consideration by another journal. It has been made publicly available as a preprint (10.1101/2024.07.05.24309978).

## Results

Among the 14,930 study participants, 2,891 (19.4%), 11,498 (77.0%), and 541 (3.6%) had high, moderate, and low overall CVH levels, respectively (Table 1). The median age of participants was 31.6 years (interquartile range [IQR]: 28.3–35.2), and the median gestational age at enrollment was 84 days (IQR: 71–103). Among participants with high, moderate, and low overall CVH levels, 380 (13.1%), 1,772 (15.4%), and 162 (29.9%), respectively, had APOs (Table 2). Pregnant women with APOs had lower overall CVH scores as well as lower scores for nicotine exposure, sleep health, BMI, blood glucose, and BP (Supplementary Table 3). As shown in Supplementary Fig. 3, CVH scores for individual components were observed to correlate positively with each other. However, significant negative correlations existed between PA and nicotine exposure, PA and sleep health, PA and BMI, and diet and blood glucose CVH scores. Gestational ages at questionnaire completion, blood sampling, and BP measurement were 191 days (median; IQR: 171–212), 136 (IQR: 113–150), and 80 (IQR: 72–87), respectively.

**Table 1:** Baseline characteristics of the complete cases in the TMM BirThree Cohort Study by overall CVH levels.

	High overall CVH	Moderate overall CVH	Low overall CVH	*P*-value
	n = 2,891	n = 11,498	n = 541	
Advanced maternal age at conception, %	655 (22.7)	3,092 (26.9)	177 (32.7)	< 0.001
Low income, %	875 (30.3)	4,237 (36.8)	269 (49.7)	< 0.001
Alcohol consumption during pregnancy, %	590 (20.4)	2,303 (20.0)	81 (15.0)	0.012
Psychological distress, %	225 (7.8)	1,237 (10.8)	100 (18.5)	< 0.001
Social isolation, %	470 (16.3)	2,286 (19.9)	154 (28.5)	< 0.001
Conception via IVF, %	110 (3.8)	611 (5.3)	33 (6.1)	0.002
Nullipara, %	964 (33.3)	4,698 (40.9)	224 (41.4)	< 0.001
GA at enrollment, day	81.0 [70.0–93.0]	84.0 [72.0–105.0]	88.0 [74.0–116.0]	< 0.001
GA at questionnaire completion, day	189.0 [170.0–208.0]	191.0 [171.0–212.0]	193.0 [176.0–214.0]	< 0.001
GA at blood sampling, day	121.0 [108.0–142.0]	138.0 [114.0–154.0]	143.0 [120.0–175.0]	< 0.001
GA at BP measurement, day	81.0 [72.0–87.0]	80.0 [72.0–87.0]	82.0 [73.0–88.0]	0.016
CVH scores				
Overall CVH score	85.0 ± 4.4	67.7 ± 7.3	44.4 ± 5.0	
Diet score	69.9 ± 26.3	44.2 ± 32.5	22.7 ± 27.5	
PA score	59.0 ± 45.3	16.7 ± 34.7	6.0 ± 21.9	
Nicotine exposure score	88.3 ± 21.0	69.3 ± 33.8	33.2 ± 34.2	
Sleep health score	83.5 ± 21.2	71.2 ± 26.6	48.5 ± 29.9	
BMI score	96.9 ± 11.3	85.8 ± 26.2	42.4 ± 32.6	
Blood lipids score	86.0 ± 22.5	65.9 ± 29.3	42.2 ± 26.2	
Blood glucose score	99.8 ± 2.5	99.3 ± 5.9	93.6 ± 17.4	
BP score	96.5 ± 10.5	89.3 ± 18.6	66.9 ± 26.6	

*CVH* cardiovascular health, *IVF* in vitro fertilization, *GA* gestational age, *BP* blood pressure, *PA* physical activity, *BMI* body mass index.

The overall CVH score was used to categorize participants into three groups: high (80–100), moderate (50–79), and low (0–49).

Data are shown as mean ± standard deviation for continuous variables and n (%) for categorical variables. For GA, data are presented as median and interquartile range.

**Table 2:** Prevalence of study outcomes by overall CVH levels.

	High overall CVH	Moderate overall CVH	Low overall CVH	*P*-value
	n = 2,891	n = 11,498	n = 541	
APOs, %	380 (13.1)	1,772 (15.4)	162 (29.9)	< 0.001
PE, %	54 (1.9)	433 (3.8)	73 (13.5)	< 0.001
GDM, %	32 (1.1)	282 (2.5)	55 (10.2)	< 0.001
PTB, %	108 (3.7)	539 (4.7)	40 (7.4)	0.001
SGA, %	213 (7.4)	718 (6.2)	31 (5.7)	0.071
LGA, %	230 (8.0)	1,155 (10.0)	103 (19.1)	< 0.001
LBW, %	225 (7.8)	912 (7.9)	44 (8.1)	0.947
NICU admission, %	100 (3.5)	531 (4.6)	37 (6.8)	0.001

*CVH* cardiovascular health, *APOs* adverse pregnancy outcomes, *PE* preeclampsia, *GDM* gestational diabetes mellitus, *PTB* preterm birth, *SGA* small for gestational age, *LGA* large for gestational age, *LBW* low birth weight, *NICU* neonatal intensive care unit.

Data are presented as n (%).

**Fig. 1:**
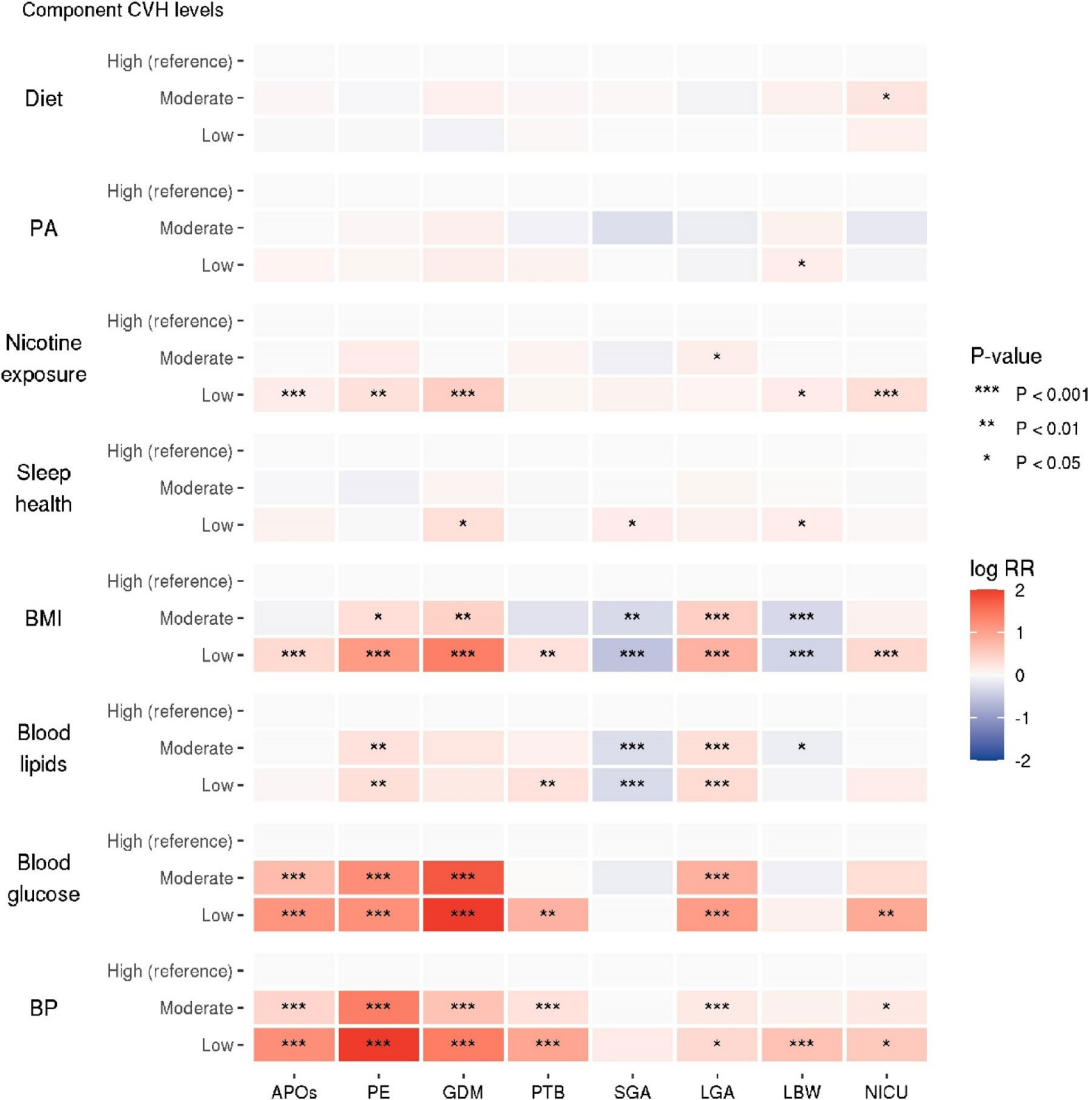
Heatmap showing associations between component CVH levels and study outcomes. The heatmap represents log transformed RRs from Poisson regression analyses with robust error variance to show the association between component CVH levels and study outcomes. Adjustments have been made for maternal age at conception, alcohol consumption during pregnancy, conception via in vitro fertilization, parity, psychological distress during pregnancy, social isolation during pregnancy, and household income. High CVH levels are used as references. Red, blue, and grey indicate positive, negative, and no association, respectively. Darker colors indicate stronger associations. The asterisk indicates the p-value. For clarity, extreme log RR values are presented as 2 or -2 when their absolute values are 2 or more. CVH, cardiovascular health; RR, risk ratio; PA, physical activity; BMI, body mass index; BP, blood pressure; APOs, adverse pregnancy outcomes; PE, preeclampsia; GDM, gestational diabetes mellitus; PTB, preterm birth; SGA, small for gestational age; LGA, large for gestational age; LBW, low birth weight; NICU, neonatal intensive care unit.

In Poisson regression analyses with robust error variance (Table 3), moderate and low CVH levels were associated with APOs with RRs of 1.15 [95% CI 1.03–1.28] and 2.14 [95% CI 1.78–2.58], respectively (*P* for trend < 0.001). Low CVH levels were also associated with a higher prevalence of PE, GDM, PTB, LGA, and NICU admission and a lower prevalence of SGA (Table 3). Using a heatmap, Fig. 1 shows the results of Poisson regression analyses with robust error variance with adjustments between eight component CVH levels, APOs, and other study outcomes. Low nicotine exposure, BMI, blood glucose, and BP CVH levels were positively associated with APOs. Low sleep health CVH levels, which were newly considered in LE8, were positively associated with GDM, SGA, and LBW, while diet and PA CVH were largely unrelated to the study outcomes. Low BMI, blood lipids, blood glucose, and BP CVH levels tended to be positively associated with the study outcomes, except that low BMI and blood lipids CVH levels were negatively associated with SGA and LBW.

**Table 3:** Results of Poisson regression analyses with robust error variance showing the association between overall CVH levels and study outcomes.

	CVH	Crude		Adjusted	
	Levels	RR [95% CI]	*P* for trend	RR [95% CI]	*P* for trend
APOs	High	Reference	< 0.001	Reference	< 0.001
	Moderate	1.17 [1.05–1.31]		1.15 [1.03–1.28]	
	Low	2.28 [1.90–2.74]		2.14 [1.78–2.58]	
PE	High	Reference	< 0.001	Reference	< 0.001
	Moderate	2.02 [1.52–2.68]		1.91 [1.44–2.53]	
	Low	7.22 [5.08–10.27]		6.53 [4.57–9.32]	
GDM	High	Reference	< 0.001	Reference	< 0.001
	Moderate	2.22 [1.54–3.19]		2.15 [1.49–3.10]	
	Low	9.18 [5.94–14.20]		8.27 [5.32–12.87]	
PTB	High	Reference	< 0.001	Reference	0.003
	Moderate	1.25 [1.02–1.54]		1.23 [1.00–1.51]	
	Low	1.98 [1.38–2.84]		1.86 [1.29–2.69]	
SGA	High	Reference	0.026	Reference	0.017
	Moderate	0.85 [0.73–0.99]		0.84 [0.72–0.98]	
	Low	0.78 [0.53–1.14]		0.75 [0.51–1.10]	
LGA	High	Reference	< 0.001	Reference	< 0.001
	Moderate	1.26 [1.10–1.45]		1.24 [1.07–1.42]	
	Low	2.40 [1.90–3.02]		2.32 [1.83–2.93]	
LBW	High	Reference	0.744	Reference	0.876
	Moderate	1.02 [0.88–1.18]		0.99 [0.86–1.15]	
	Low	1.05 [0.76–1.44]		0.98 [0.71–1.36]	
NICU admission	High	Reference	< 0.001	Reference	0.002
	Moderate	1.34 [1.08–1.65]		1.28 [1.03–1.59]	
	Low	1.98 [1.36–2.88]		1.82 [1.24–2.66]	

*CVH* cardiovascular health, *APOs* adverse pregnancy outcomes, *RR* risks ratio, *CI* confidence interval, *PE* preeclampsia, *GDM* gestational diabetes mellitus, *PTB* preterm birth, *SGA* small for gestational age, *LGA* large for gestational age, *LBW* low birth weight, *NICU* neonatal intensive care unit.

RRs and 95% CIs were estimated using Poisson regression analyses with robust error variance with high CVH levels used as a reference.

Maternal age at conception, alcohol consumption during pregnancy, conception via in vitro fertilization, parity, psychological distress during pregnancy, social isolation during pregnancy, and household income were included in the adjusted models.

**Fig. 2:**
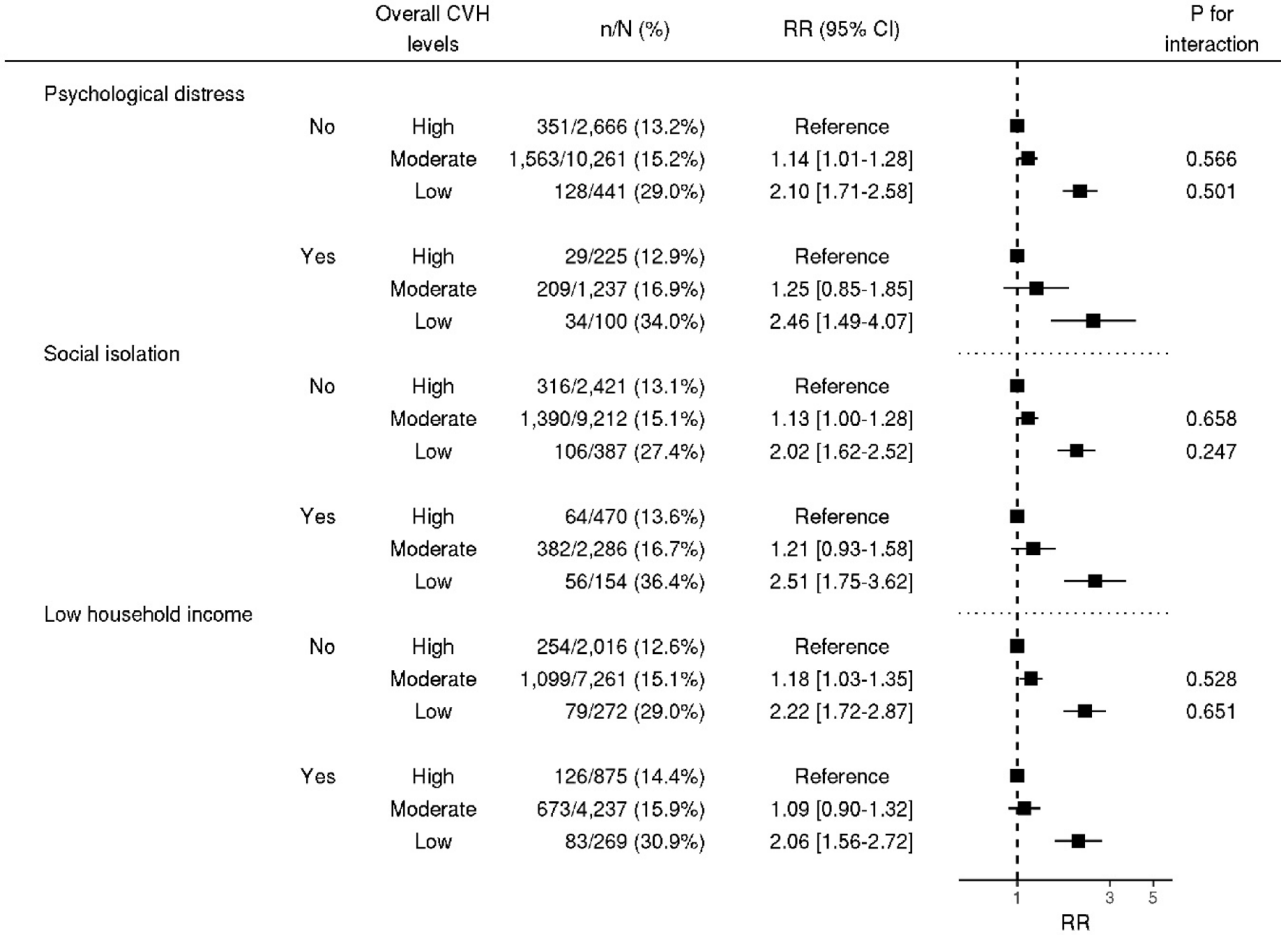
Subgroup analyses by psychological distress, social isolation, and income. RRs and 95% CIs are calculated using Poisson regression analyses with robust error variance to show the association between overall CVH levels and APOs, with adjustment for maternal age at conception, alcohol consumption during pregnancy, conception via in vitro fertilization, parity, psychological distress during pregnancy, social isolation during pregnancy, and household income. The "n/N (%)" indicates the number of cases and the total number in that stratum and its ratio. CVH: cardiovascular health; RR: risk ratio; CI confidence interval.

**Fig. 3:**
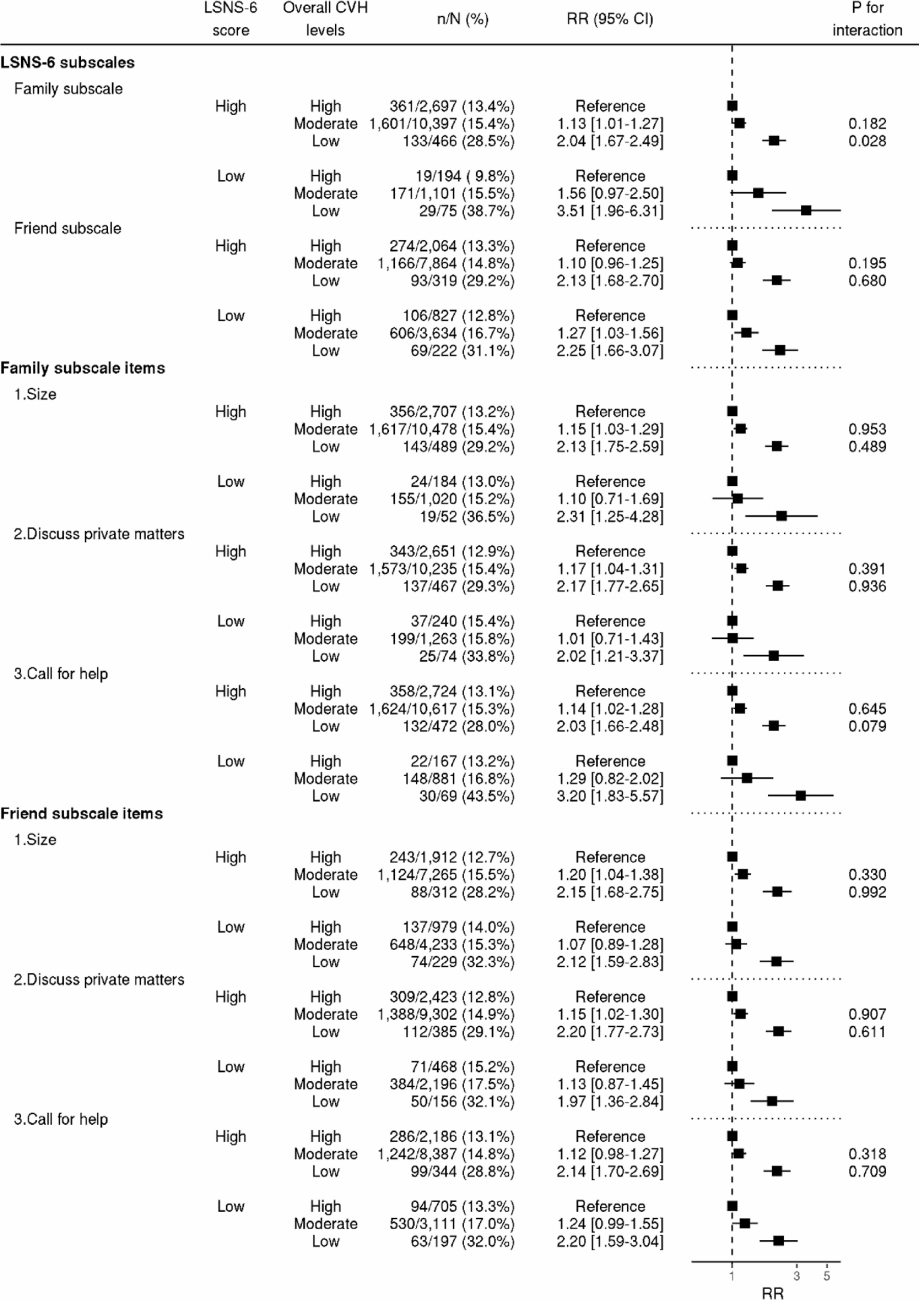
Subgroup analyses by subscales and each item in LSNS-6. RRs and 95% CIs are calculated using Poisson regression analyses with robust error variance to show the association between overall CVH levels and APOs, with adjustment for maternal age at conception, alcohol consumption during pregnancy, conception via in vitro fertilization, parity, psychological distress during pregnancy, social isolation during pregnancy, and household income. The "n/N (%)" indicates the number of cases and the total number in that stratum and its ratio. The details of LSNS-6 are presented in Supplementary Table 2. LSNS, Lubben Social Network Scale; CVH, cardiovascular health; RR, risk ratio; CI, confidence interval.

Subgroup analyses of psychological distress, social isolation, and income demonstrated that low overall CVH levels had a stronger association with APOs in socially isolated participants, although the interaction was not statistically significant (P for interaction = 0.247). RRs for APOs were 2.10 [95% CI 1.71–2.58] and 2.46 [95% CI 1.49–4.07] for participants with and those without social isolation, respectively (Fig. 2). Among pregnant women with low CVH levels, the prevalence of APOs at delivery was higher among socially isolated pregnant women (36.4% vs. 27.4%); however, this difference was attenuated among pregnant women with high CVH levels (13.6% vs. 13.1%). Subgroup analyses of each item and subscale of social isolation were conducted, demonstrating that low overall CVH levels were more strongly associated with APOs in participants with a low family subscale score (P for interaction = 0.028). RRs for APOs were 2.04 [95% CI 1.67–2.49] and 3.51 [95% CI 1.96–6.31] for participants with a family subscale score of ≥ 6 and those with a score of < 6, respectively (Fig. 3). Analyses of each of the LSNS-6 items, on the family and the friend subscale, showed a significant interaction between overall CVH levels and the “Call for help” item in the family subscale.

Sensitivity analyses showed results that were largely consistent with those of the main analyses (Supplementary Figs. 4–6).

## Discussion

### Principal findings

Low overall CVH levels were associated with a higher prevalence of APOs, PE, GDM, PTB, LGA, and NICU admission and a lower prevalence of SGA. The association of low overall CVH levels with APOs was stronger for socially isolated pregnant women than for pregnant women who were not socially isolated, although the interaction was not statistically significant. Among the LSNS-6 items and subscales, participants with low family subscale scores and those who reported having fewer close relatives to whom they could call for help were more affected by low overall CVH levels.

### Results in the context of what is known

Two previous studies investigated the relationship between poor CVH and APOs^19,20^. The first study^19^ calculated the overall CVH status using only five components and reported associations with PE, unplanned cesarean section, LGA, sum of skinfolds, and insulin sensitivity. The second study^20^ calculated the overall CVH status using all eight components of LE8 and demonstrated associations with LGA, and macrosomia. Our results extend these findings and further illustrate the link between overall CVH, represented by all LE8 components, and clinical outcomes such as PE, GDM and NICU admission. In the previous studies^19,20^, high overall CVH levels were positively associated with SGA, primarily through the BMI component. This counterintuitive finding led to a clinical misinterpretation that “high CVH is a risk factor for SGA.” This is explained by a shift in the birth weight distribution among mothers with poor CVH, due to maternal overnutrition or metabolic dysregulation, leading to a reduced prevalence of SGA and an increased prevalence of LGA^41^. However, this intrauterine overnutrition may cause metabolic alterations in the offspring that are not captured by birth weight alone and could result in adverse birth consequences^42^. Indeed, our study reported similar results but also showed that a low overall CVH level is a risk factor for APOs and NICU admission. Taken together, our findings support the integration of CVH in antenatal care. Furthermore, the association between sleep health, a new component of LE8, and study outcomes such as GDM, SGA, and LBW suggests that LE8 may be more practical than LS7.

Our study demonstrated a more pronounced association between low overall CVH levels and APOs in socially isolated pregnant women than in pregnant women without social isolation. Negative social determinants of health, including social isolation, are well-established risk factors for CVD and mortality in non-pregnant people^43,44^ and have been identified to influence CVH in pregnant women^45^. However, the interaction between these factors and CVH remains elusive. Previously, a study^21^ involving middle-aged and older adults, predominantly Europeans, showed that life expectancy was comparable across socioeconomic strata among participants with optimal CVH but not among those with poor CVH, suggesting that improved CVH could mitigate health inequalities. Our findings are consistent with previous results indicating that pregnant women have a similar risk of APOs regardless of their social isolation status when their CVH improves, underscoring the importance of providing support to this population. Furthermore, our findings suggest that socially isolated pregnant women, particularly those with limited family relationships or fewer close relatives to whom they can call for help, may be more vulnerable to the impact of low CVH. Beyond the general concept of "social isolation," it may be necessary to consider the specific social conditions of pregnant women.

Notably, psychosocial distress during pregnancy did not significantly modify the effect of CVH, suggesting that psychological distress and CVH were additively related to APOs. Previous studies have reported a bidirectional relationship between CVH and psychological status, identifying maternal psychological distress as a possible risk factor for poor CVH during pregnancy^46^. In addition, improvements in CVH from early pregnancy to 6 months postpartum have been linked to reduced postpartum depressive symptoms^47^, and psychological distress has been found to be associated with APOs^48–50^. Therefore, a simultaneous assessment and consideration of both aspects in clinical practice is recommended.

### Clinical implications

Assessment of CVH as undertaken by our study has potential implications for clinical practice. Importantly, there is a need to adopt a comprehensive approach to health assessment rather than focusing solely on a specific aspect of health status. Utilizing an index of overall CVH instead of a single CVH component enables clinicians to address complex interactions among risk factors, such as smoking cessation that may lead obesity or exercise habits that may impact the duration of sleep. In particular, for socially isolated pregnant women who demonstrated a heightened vulnerability to low CVH in this study, prioritized access to primary care, lifestyle education, and access to pharmacotherapies is essential. Regardless of social isolation status, the risk of APOs was consistently low in the group with high CVH levels, suggesting that increasing CVH levels in pregnant women may reduce health disparities.

## Research implications

Considering the correlation between CVH before and that during pregnancy^51^, incorporation of LE8 assessment in conception planning may help prevent future APOs. LE8 advocates improvements by lifestyle modification or the use of pharmacotherapy, empowering women to proactively enhance their CVH in preparation for pregnancy. We anticipate that further studies will reveal the relationship between pre-conception CVH status and APOs.

### Strengths and limitations

Notably, our study population was approximately five times larger than that in prior studies^19,20^ that investigated the associations between CVH and APOs. Another strength of this study lies in the inclusion of pregnancy outcomes, providing a comprehensive assessment of the clinical importance of CVH. Also, our sensitivity analyses reinforced the robustness of the results.

Despite these strengths, our study had some limitations. First, the timing of CVH measurements varied among participants, although all assessments, including questionnaires, blood pressure measurements, and blood samples, were conducted during pregnancy. Consequently, the point at which CVH is most strongly associated with APOs remains unclear. Second, the course of pregnancy may influence behavior during pregnancy, introducing the possibility of reverse causation. Sensitivity analyses in participants with blood samples collected before or at 20 weeks showed results consistent with those of the main analyses, suggesting limited reverse causality related to glucose and lipid status. However, the effects of other conditions, such as PE diagnosis and fetal growth restriction, remain uncertain. Finally, the validity of the LE8 in the Japanese population has not yet been established. Population differences and variations in LE8 definition, such as the use of the 8-item Japanese Diet Index vs. the 16 items of Mediterranean Eating Pattern for Americans, may impact the accurate assessment of CVH by LE8.

## Conclusion

In conclusion, CVH status may be a useful index for evaluating the risk of APOs. Socially isolated pregnant women may be more vulnerable to the effects of low CVH status.

## Electronic supplementary material

Below is the link to the electronic supplementary material.

Supplementary Material 1

## Data Availability

Individual data is available upon request to the corresponding author after the approval of the Ethical Committee and the Materials and Information Distribution Review Committee of Tohoku Medical Megabank Organization.
